# Cost-effectiveness of internet-based cognitive behavior therapy for irritable bowel syndrome: results from a randomized controlled trial

**DOI:** 10.1186/1471-2458-11-215

**Published:** 2011-04-07

**Authors:** Erik Andersson, Brjánn Ljótsson, Filip Smit, Björn Paxling, Erik Hedman, Nils Lindefors, Gerhard Andersson, Christian Rück

**Affiliations:** 1 Department of Clinical Neuroscience, Stockholm Center for Psychiatry Research, Karolinska Institutet, Stockholm, Sweden; 2 Department of Epidemiology and Biostatistics, EMGO Institute for Health and Health Care Research, VU University Medical Centre, Amsterdam, Netherlands; 3 Centre of Prevention and Early Intervention, Trimbos Institute (Netherlands Institute of Mental Health and Addiction), Utrecht, Netherlands; 4 Department of Behavioural Sciences and Learning, Swedish Institute for Disability Research, Linköping University, Sweden

**Keywords:** Cognitive behavior therapy, internet, IBS, cost-effectiveness analysis

## Abstract

**Background:**

Irritable Bowel Syndrome (IBS) is highly prevalent and is associated with a substantial economic burden. Cognitive behavior therapy (CBT) has been shown to be effective in treating IBS. The aim of this study was to evaluate the cost-effectiveness of a new treatment alternative, internet-delivered CBT based on exposure and mindfulness exercises.

**Methods:**

Participants (N = 85) with IBS were recruited through self-referral and were assessed via a telephone interview and self-report measures on the internet. Participants were randomized to internet-delivered CBT or to a discussion forum. Economic data was assessed at pre-, post- and at 3-month and 1 year follow-up.

**Results:**

Significant cost reductions were found for the treatment group at $16,806 per successfully treated case. The cost reductions were mainly driven by reduced work loss in the treatment group. Results were sustained at 3-month and 1 year follow-up.

**Conclusions:**

Internet-delivered CBT appears to generate health gains in IBS treatment and is associated with cost-savings from a societal perspective.

## Background

Irritable bowel syndrome (IBS) is a highly prevalent gastrointestinal disorder [1] that is associated with production losses [2,3] and increased levels of health service utilization [3,4]. The high prevalence rate combined with the costs associated for each afflicted individual makes IBS a considerable economic burden for society. Research indicates that psychological interventions - such as cognitive behavior therapy (CBT), hypnotherapy and psychodynamic therapy - can be effective in reducing IBS symptoms [5]. In addition, there is some evidence indicating that psychological treatments are cost-effective. In a study by Creed et al. [6], 171 patients with IBS were randomized to receive either 8 sessions of individual psychodynamic therapy, paroxetine, or care as usual. At one-year follow-up, the psychotherapy condition was associated with significant reductions in health care consumption when compared to care as usual, whereas the paroxetine group did not show a similar reduction. In a study by McCrone et al. [7], CBT was found to have a reasonable short-term cost-effectiveness but not beyond 3-month follow-up. In summary, although psychological treatments are effective in reducing IBS symptoms there is insufficient evidence of their cost-effectiveness.

Our research group has recently conducted a randomized controlled trial of internet-delivered CBT for IBS [8]. The main reason for using the internet as method of delivery is to increase the availability of the treatment. Evidence-based psychological treatments are often unavailable due to a lack of properly trained therapists [9]. Internet-delivered CBT with minimal therapist contact has been found to be effective for a number of psychiatric and somatic problems [10,11], and are likely to be cost-effective and more accessible to patients [12]. However, there is little evidence regarding the economic aspects of internet-delivered CBT.

## Methods

### Sample

Participants were eligible for the study if they had been diagnosed with IBS by a physician before applying for the study and if they presently fulfilled the Rome III criteria for IBS [13]. The Montgomery Åsberg Depression Rating Scale - Self report [MADRS-S; [14]] was used to exclude participants, fulfilling criteria for suicide ideation (item 9 ≥ 4), and severe depressive symptoms(total score ≥ 30). Participants fulfilling criteria for substance dependence according to Alcohol Use Disorders Identification Test [AUDIT; [15]] or Drug Use Disorders Identification Test [DUDIT; [16]] and participants suffering from psychosis, manic episode, or anorexia were also excluded.

#### Procedure

**Figure 1:**
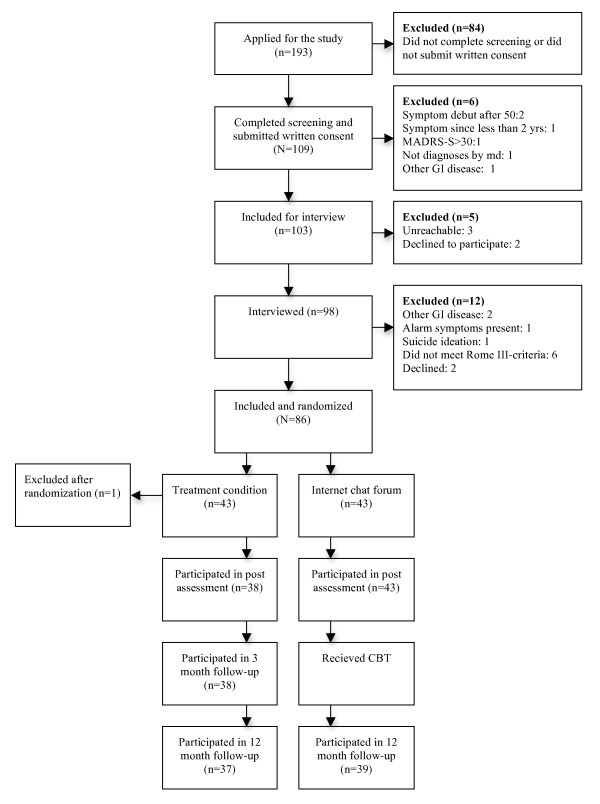
**Flowchart of study participants**. (no figure legend).

Figure 1 displays participant flow through the selection procedure, randomization and assessments. The participants were recruited through self-referral from the general adult population in Sweden. A total of 193 individuals applied for the study, 98 were interviewed and 86 were included for randomization. Telephone interviews were conducted by graduate psychology students or psychologists and included structured questions about Rome III-criteria and also the Mini-International Neuropsychiatric Interview [MINI; [17]] to assess any psychiatric co-morbidity that could be cause for exclusion. All interviews were reviewed by the study's gastroenterologist who made the final decision if a participant should be included in the study. After having been randomized to the treatment condition one participant underwent a colostomy and was subsequently excluded from the study and all further data analysis. Demographics for the study participants are displayed in Table 1.

**Table 1:** Demographics

	Total (n = 85)	CBT (n = 42)	Control (n = 43)
**Sex**			
Male (*n)*	13	7	6
Female (*n)*	72	35	37
**Age**			
mean years (*sd*)	34.6 (9.4)	36.4 (10.1)	32.8 (8.6)
min-max years	20-61	21-61	20-50
**Years since diagnosis**			
mean years (*sd*)	6.3 (7.3)	7.2	5.5
min-max years	0-41	0-41	0-22

The study included pre- and post-treatment assessments for the treatment condition. Follow-up data were collected at 3-month follow-up for the treatment condition. After ten weeks, the participants in the control condition were crossed over to the active treatment. Follow-up data were also collected for all participants in the study approximately one year (15 to 18 months) after receiving the treatment.

The study protocol was approved by the regional ethics committee in Stockholm, Sweden.

### Interventions

The experimental group was given a ten-week internet-delivered cognitive behavioral treatment with therapist support via e-mail. The mean therapist time spent on each participant was 165 minutes (SD = 85 min). The treatment consisted of graded exposure to IBS-symptoms and mindfulness exercises. The treatment protocol is further described in Ljótsson et al. [18] and Ljótsson et al. [8]. Participants in the control group took part in a discussion forum and could contact a therapist for general support if they wished (mean therapist time was 10 minutes, SD = 23).

### Clinical outcome

The primary outcome was the Gastrointestinal Symptom rating scale-IBS version (GSRS-IBS) [19]. A clinically significant improvement was defined as a 50% reduction of GSRS-IBS score [20].

### Cost assessment

Cost data were obtained with the Trimbos and Institute of Medical Technology Assessment Cost Questionnaire for Psychiatry (TIC-P) [21]. In this questionnaire, participants report their health care consumption during the last month (e.g. GP visits), as well as time spent in informal health enhancing activities (e.g. self-help groups and informal care from friends). Participants also report their level of sick leave and reduced work capacity both at work and in the domestic realm and unemployment status during the last month.

These self-reports were used to estimate the costs of each participant's health care consumption, work loss, and work cutback. Medication costs were based on the free market price in Sweden. Costs from health service visits were estimated using established tariffs in Sweden. The human capital approach was used for valuation of the productivity losses, which means that monetary losses associated with work loss and work cutback were based on the participants' gross earning for the full length of their sick leave [22]. Gross earnings were estimated using the average salary in Sweden by education level. Domestic losses were estimated at $ 13.17 per hour, reflecting the free market price of domestic help [23]. The costs were originally computed in Swedish Crowns and then converted into US $ using the purchasing power parities of the OECD for the reference year 2008 [24].

The direct medical costs associated with the intervention were mainly driven by the costs of therapists. In this study, the therapists were graduate psychology students under supervision, but in the analysis we used the full economic cost price of a licensed clinical psychologist.

The time the therapist spent on treating the participants was registered and multiplied by this cost. We also estimated the costs of the participants' time, again at $13.17/hour, thus equating it with domestic help and assuming that people engaged in the intervention after office hours. The cost estimations of the control group were based on the time spent on the internet discussion forum. The cost estimations in the treatment group were based on the self-reported time the participants spent reading the treatment material and performing homework exercises plus the time spent on the discussion forum,

### Analysis

The analysis was conducted in accordance with the intention to treat principle, which means that all participants were included in analysis regardless of adherence to treatment. Missing data were imputed using the last observation carried forward method. The statistical analysis was conducted in four steps using STATA IC/11.0 (Stata Corp).

First, the cost differences between the treatment and control groups were estimated at pre-treatment and post-treatment. All costs were extrapolated to a 12-month period. Since the cost data were assumed to be non-normally distributed, *p*-values were estimated using a general linear model while employing non-parametric bootstrap analysis (5,000 replications). Such analyses are considered to generate robust estimates of standard errors of the costs [25].

Secondly, we compared the counts of clinically significant improvements across the conditions. Using a Poisson regression framework allowed us to test if the rate of favorable treatment responses in the experimental condition was higher than 1 relative to the control condition. The number needed treat (NNT) was computed as the inverse of the risk difference, which was obtained under a linear probability model [26].

**Figure 2:**
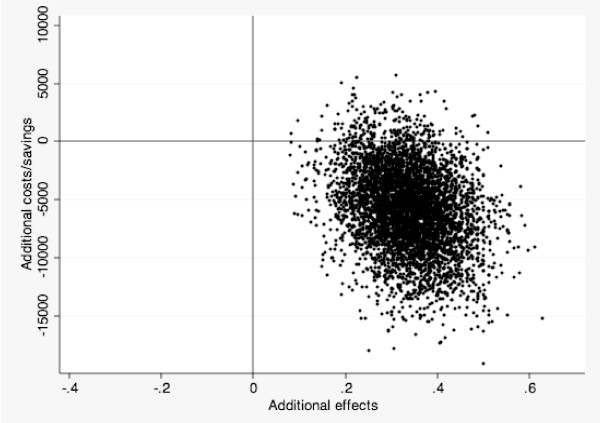
**Cost-effectiveness plane**. (no figure legend).

Thirdly, the incremental cost-effectiveness ratio (ICER) was estimated. We computed pre-post differences in costs and effects. We then computed differences in costs and effects between both conditions taking the cost difference over the effect difference: (C(exp) - C(ctr))/(E(exp) - E(ctr)). C is the difference of the costs of IBS and the intervention between the pre and post assessments. E refers to the treatment response between both conditions [22]. This calculation was repeated 5,000 times (for each bootstrap sample) generating a scatter of simulated ICERs across the ICER plane (see Figure 2).

Fourthly, the robustness of the results was tested in four different sensitivity analyses. First, the main analysis was repeated with only the direct medical costs included, hence narrowing the economic evaluation to a health service perspective. Second, the total costs were calculated with the unemployment costs excluded. This was done because the costs due to productivity losses from unemployment are large when accumulated over a 12-month period and could (in spite of randomization) be affected by factors not associated with IBS. Third, the missing data were reanalyzed using linear regression imputation as implemented in STATA. This was done to test whether last observation carried forward was indeed a more conservative imputation method. Fourth, we investigated the long-term impact on the ICER using 3-month and 1-year follow-up economic data. Since the control group was crossed over to treatment there was no comparison group at the follow-up assessment. The 3-month and 1 year follow-up of the treatment group data were therefore compared with the post-treatment data of the control group, thus assuming that the control group would have been unchanged if had been untreated during the follow-up periods. This was done to test whether the extrapolation to a 12-month period was a reliable analysis procedure.

Lastly, since the control group was crossed over to treatment and also participated in the follow-up, the long-term costs were calculated separately for that group.

## Results

### Costs

Table 2 presents the annual per capita costs of IBS at pre-treatment and post-treatment assessment for both treatment and control groups and 3-month and 1 year follow-up for the treatment group. At post treatment, we found significant cost differences regarding the gross total monetary change, Z = -2.06, *P *< 0.05, as well as the indirect non-medical costs, Z = -2.20, *P *< 0.05.

**Table 2:** Mean annual costs

	Annual per capita cost ($)					
	Pre treatment		Post treatment		3-month follow-up	12-month follow-up
	**CBT**_**(SE)**_	**Control**_**(SE)**_	**CBT**_**(SE)**_	**Control**_**(SE)**_	**CBT**_**(SE)**_	**CBT**_**(SE)**_
Direct medical costs	3,580 (757)	3,241 (706)	3,222 (756)	3,547 (790)	2,096 (445)	1,754 (450)
Health care visits	3,444 (756)	3,053 (670)	3,153 (760)	3,323 (746)	1,895 (435)	1,666 (443)
Medications	136 (19)	188 (70)	69 (18)	225 (73)	38 (8)	88 (26)
Direct non-medical costs	1,045 (370)	1,086 (498)	958 (449)	571 (305)	735 (330)	371 (131)
Indirect non-medical costs	16,241 (2893)	10,709 (2308)	11,227 (2544)	12,619 (2482)	11,324 (2478)	13,677 (2906)
Workloss	11,934 (1668)	6,167 (1295)	10,086 (2567)	9,674 (2505)	9,561 (2511)	12,314 (2951)
Work cutback	2,919 (864)	3,545 (943)	1,097 (292)	2,945 (734)	1,262 (323)	1,362 (402)
Domestic	1,388 (301)	997 (181)	921 (225)	1102 (300)	500 (117)	529 (168)
Total (excl. intervention costs)	20,867 (3395)	15,036 (2615)	15,407 (3048)	16,737 (2713)	14,156 (2660)	15,802 (3023)
Intervention costs			1,580 (158)	135 (26)	1,580 (158)	1,580 (158)
Total (incl. intervention costs)	20,867 (3395)	15,036 (2615)	16,988 (3068)	16,872 (2720)	15,736 (2645)	17,382 (3038)

### Treatment efficacy

The fraction of recovered participants in the intervention condition at post treatment was 15/42 = 0.36 and 1/43 = 0.02 in the control condition. Hence, the likelihood ratio for a favorable treatment response was 0.36/0.02 = 15.36, representing a 15-fold increase of the recovery rate in the intervention relative to the control condition (95% confidence interval 2.1 to 111.1). This was statistically significant, Z= 2.64, *P *< 0.01. The NNT to generate one clinically significant improvement was 2.99 (95%CI 2 to 5), which means that three patients need to receive the intervention to generate one clinically significant improvement. At the 3-month follow-up, the fraction of recovered participants in the CBT condition was 14/42 = 0.33, and at one-year follow-up this figure was 18/42 = 0.43. In sum, the effects appear to be sustained over a 12-month period.

### Cost effectiveness

The cost change for the treatment group was 16,988 - 20,867 = -3879. The cost change in the control group was 16,872 - 15,036 = 1835. The incremental cost effectiveness ratio (ICER) was (-3879 - 1835)/(0.36 - 0.02) = -16,806. This means that each significant clinical improvement in IBS is associated with a societal cost-reduction of $16,806. Figure 2 presents the scatter of simulated ICERs across the four quadrants of the ICER plane. If the bulk of simulated ICERs appear in the southeast quadrant of the figure, lower costs are associated with a health gain. From a cost-effectiveness perspective, this is the most favorable outcome. If a majority of the simulated ICERs appear in the northwest quadrant, higher costs are associated with a lowered effectiveness, thus making the new intervention unacceptable from a cost-effectiveness perspective. Nearly all simulated ICERs (96%) were located in the southeast quadrant, indicating that the treatment produced beneficial effects and reduces costs for society compared to a no treatment control condition.

### Sensitivity analyses

When only including direct medical costs in the estimation, 48% of the simulated ICERs were located in the southeast quadrant. When analyzing all data but excluding the unemployment variable, 41% of the dots were located in the southeast quadrant. Data were reanalyzed using regression imputation of missing data instead of last observation carried forward (4 imputations, R-square = 0.93). A majority (98%) of the simulated ICERs were still located in the southeast quadrant, attesting to the robustness of our findings. Finally in order to investigate the long-term impact, we repeated the analyses with follow-up economic data for the treatment group. Using 3-month and 1-year follow-up data, 97% and 89% of the dots were located in the southeast quadrant, respectively.

### The control group at follow up

After the post-treatment the control group was crossed over to treatment. At the 1 year follow-up the group also presented with cost reductions (pre treatment; $15,036, follow-up; $13,008). Additionally 19/43 of the control group participants reported clinically significant symptom relief.

## Discussion

The aim of this study was to determine whether internet-delivered CBT would be a cost-effective or perhaps even a cost-saving intervention for IBS. The results give preliminary evidence of the internet-delivered treatment as a cost-effective way of offering treatment; better still, our observations suggest that the intervention is cost saving from a societal point of view. Participants in the treatment condition demonstrated significant reductions in IBS symptoms compared to the control condition. These improvements were accompanied by cost reductions at post-treatment and at follow-up. While the intervention did introduce some costs of its own, these costs were offset by greater productivity levels both at paid work and in the domestic realm. Thirty-six percent of the participants in the treatment condition were clinically improved and for each case of clinical improvement in IBS symptoms $16,806 of societal costs were saved. Irrespective of modeled scenario and choice of imputation method the online CBT intervention was associated with cost offsets, demonstrating robustness of our findings. The control group showed similar improvements and cost-savings after being crossed over to treatment.

The results from this trial differ from the McCrone et al. [7] and Creed et al. [6] studies. In the Creed et al. study, significant reductions in health care costs were found at the one-year follow-up but no significant reductions in work loss for the psychotherapy group. One possible explanation for this difference could be that this study used a broader perspective including both work cutback as well as domestic loss in the cost-effectiveness analysis. In the McCrone et al. study, the CBT group did not show any significant reductions in work loss and the treatment was not cost-effective beyond 3-month follow-up. In the original treatment study that the McCrone et al. report was based on, the participants' improvements in symptom actually declined after the 3-month follow-up [27]. In contrast, the participants in our study demonstrated long-term maintenance of symptom reduction that could explain the long-term cost effectiveness. By and large, the results from this study provide additional evidence for internet-delivered CBT as a cost-effective treatment alternative for IBS.

Our study has several limitations. First, the cost data was collected using self-reports and the accuracy of this methodology can be open-to-question. However, the recall period was relatively short (4 weeks) and there is no reason to assume that inaccuracies overly bias any of the two treatment conditions. In addition, previous research studies have indicated that self-reports are as reliable as administratively recorded data [28]. The TIC-P has been used in numerous studies [29-31] and has the advantage of being a broad measure covering health care uptake and productivity losses both in paid work as well as in the domestic realm. Therefore, the self-report methodology could be regarded as a feasible choice of measurement. Secondly, the use of 50% decline in IBS symptoms measured as criterion for clinically significant improvement might be inappropriate. This criterion has been used in several trials [20] of psychological treatment but other measures of clinically significant improvement have been proposed. The participants' subjective experience of "adequate relief" [32] has been used in both pharmacological [33] and psychological trials [9]. A 50 point reduction in score on the IBS-SSS [34] has also been used as clinically significant improvement in symptoms. These differences in determining a clinically significant outcome are problematic as they make it difficult to compare trials and treatments. It would therefore have been preferable if at least one of the adequate relief and IBS-SSS measures had been included in this study. Thirdly, the control condition participants were randomized to a waiting list and were therefore aware that they would eventually receive the treatment. This factor could possibly have an impact on the cost-effectiveness results since the study design did not allow control for attention and expectancy of improvement. A proper attention control condition would have provided a more reliable estimate the cost-effectiveness of the specific treatment used. Fourthly, the calculations are based on internet CBT in a research setting and the real market price of such interventions is still unknown. Fifth, we did not directly ask the participants about their gross earning but instead used the average Swedish salary based on the participant's education level. This lack of information could possible produce errors in the mean cost calculations regarding production losses. However, the participants were randomized and potential errors should not affect the between group ICER estimations. Finally, as the control group was crossed over to treatment no comparison group was available at the 3-month and 1 year follow-up. The short experimental time period makes the extrapolated estimates of long-term cost-effectiveness somewhat less reliable. However, when repeating the analyses with 3-month and 1 year follow-up clinical data, the scatter of the simulated ICERs over the cost-effectiveness plane did not differ in any substantial way and would not change our conclusions.

## Conclusions

To summarize, the findings from this study indicate that internet-delivered CBT for IBS can both be effective in treating IBS and generate cost savings for the society. Future evaluations should continue to investigate the cost-effectiveness as well as look more closely at the economic ramifications with regard to favorable treatment response. More research is also needed to expand our knowledge concerning the economic impact of internet-delivered CBT for IBS. We recommend that future studies use a more potent placebo condition and longer follow-up periods.

## Competing interests

The authors declare that they have no competing interests.

## Authors' contributions

EA was main responsible in interpreting health economic data and performed all statistical analyses. He also performed psychiatric interviews in the study and was responsible for drafting this manuscript. BL was the study project manager. He wrote the treatment manual and participated in the drafting of this manuscript. FS participated in the interpretation of health economic data and statistical analyses. He also contributed to the drafting of the manuscript. BP crosschecked all health economic data and participated in the statistical analyses. He also contributed to the drafting of the manuscript. EH participated in the conception of the study and in revising this manuscript. NL participated in the study conception, its design and management, analysis of data, interpretation and revising this manuscript. GA participated in the study conception, its design and management, analysis of data, interpretation and revising this manuscript. CR participated in the conception of the study and its design, was responsible for psychiatric assessments and in the supervising of this manuscript. All authors read and approved the final manuscript.

## Pre-publication history

The pre-publication history for this paper can be accessed here:

http://www.biomedcentral.com/1471-2458/11/215/prepub
